# A study on the effect of school culture on teachers’ commitment to curriculum implementation: The mediating role of self-efficacy and job satisfaction

**DOI:** 10.1016/j.heliyon.2024.e29183

**Published:** 2024-04-04

**Authors:** Azzeddine Boudouaia, Abdo Hasan AL-Qadri, Asma Houichi, Salma Diafi

**Affiliations:** a College of Education, Zhejiang University, China; b School of Humanities and Education, Xi'an Eurasia University, China; c Teachers Training College Bouzareah (ENSB), Algeria; d School of Psychology, Central China Normal University, China

**Keywords:** Commitment to curriculum implementation, Job satisfaction, School culture, Self-efficacy, Teachers

## Abstract

Within the realm of English as a Foreign Language (EFL) instruction, there was pressure and drive to achieve success in the execution of the curriculum. Nevertheless, teachers encountered difficulties in implementing the curriculum and achieving successful outcomes. In order to achieve the desired objectives in the implementation of the EFL curriculum, it is necessary to have positive interacting variables that contribute to the overall dynamics. This study aimed to investigate the effect of school culture on English language teachers' commitment to curriculum implementation (TCCI), as well as the mediating role of external factors, self-efficacy (SE), and job satisfaction (JS) on this effect. The study sample involved 533 English language teachers from public middle schools in Algeria. A questionnaire was adopted for teacher-principal relationship (TPR), teacher-teacher relationship (TTR), teacher-student relationship (TSR), SE, JS, and TCCI. The analysis was done with the help of SPSS 26, JASP, and structural equation modeling (SEM). The results showed a significantly positive direct effect of TPR, TTR, and TSR on TCCI. Furthermore, there was a positive indirect effect of TPR, TTR, and TSR had a positive indirect effect on TCCI via SE and JS. These findings suggested a number of different courses of action for policy, research, and practice regarding teachers' commitment to curriculum implementation and the interaction between teachers, principals, and students over the next several years to raise teachers’ SE and JS levels.

## 1 Introduction

Pedagogical changes resulting from curricular reform over the last several decades [1] have revolutionized the field of teaching English as a foreign language (EFL). As a result, nowadays, teachers have more options than ever before when it comes to the method of delivery that may best promote the efficient implementation of new curricula. In addition, several adjustments can take place in the ways that language courses are taught, assessed, and managed. These resources can enhance and enrich teachers' classroom experiences, impacting regions, countries, schools, and educators to varying degrees [2,3].

In EFL education, there was tremendous pressure and motivation to become fruitful in curriculum implementation. In developing countries such as Algeria, in the context of this study, the new curriculum, which is based on a competency-based approach and was undertaken in middle schools in 2016, of which the English language curriculum was among its parts, was poorly implemented by teachers [4]. Unfortunately, despite the fact that most of the current EFL teachers experience the old and current curricula, teachers are struggling with putting into practice the current curriculum and establishing fruitful achievement of implementation success scores [4,5]). Since 2016, there has been an increasing necessity for new actions and activities among EFL teachers. Year after year, many of them found themselves unable to complete several courses and the drive to attain curriculum goals [3]. This was depicted in the Educational, Scientific, and Cultural Organization (UNESCO) report in 2017. UNESCO placed Algeria 119th out of 140 Arab and international nations in terms of educational quality [5]. A question that appeared here was whether EFL instructors are aware of the causes of a lack of commitment to curriculum implementation. Despite the possibility of influential factors, TCCI played a crucial role in the education system. It was critical to have an in-depth understanding of the factors that influence EFL TCCI in order to guide practice in similar situations and settings and to prepare for potential uncertainty.

The Social Cognitive Theory (SCT) was adopted in this study to explain the factors that influence TCCI. SCT has been proven to help determine the factors that affect teachers’ instruction [6]. The SCT stands out as one of the popular theories being extensively validated across different educational contexts when explaining TCCI. However, there was a need to further test the generalization of SCT for EFL curriculum implementation.

Regarding SCT, environment, personality, and behavior are three main interactive factors that have triadic and asymmetric relationships that determine the performance of an individual [6]. In EFL curriculum implementation, to accomplish the desired aims, the dynamics of helpful interactive factors have to exist. These factors were related to the teacher and the surrounding environment, i.e., internal and external. In fact, when teachers keep reminding themselves that implementation comprises putting into practice the curriculum principles for learners attempting to grow up, For successful implementation, these factors must be at the core, with supportive external (environment) and internal (personality) factors surrounding them [7]. The literature suggests that external factors generally revolve around the school culture [[8], [9], [10], [11]]. School culture was prominent in teachers' curriculum implementation to the extent that if they were working against the implementation, the teacher would not be clear about their practices [7]. School culture consists of the relationship between agents, i.e., the relationship between school principal and teacher, teacher and teacher, and teacher and students. That is to say, what teachers should do specifically to manage the rapport with school principals, colleagues, and students is necessary to ensure successful instruction [12,13]. Without this sociological sympathy, teachers may not be able to understand how to approach the curriculum implementation process. Bearing in mind the prominence of the relationship between school teacher-principal, teacher-teacher, and teacher-student in curriculum implementation, it has been found that there have been limited studies that have dealt with such an issue [3]. Previous studies have focused on teachers relationship with the curriculum through professional and policy context [14], TSR and student engagement [15,16], TSR quality [17], teacher-principal relationship and teachers' professional well-being [18], TPR and leadership [19], and teacher-principal and students’ academic outcomes [20]. To fill this gap in the literature, it became vital to delve into the effect of TPR, TTR, and TSR on EFL TCCI.

Furthermore, a growing body of research into EFL teachers' internal factors (personality) suggests that SE and JS are two critical factors that shape curriculum implementation [11]. However, teachers' SE and satisfaction may also relate to other factors to depict their effect on the EFL curriculum implementation. Researchers have focused on SE and performance [21,22], efficacy beliefs and JS [[23], [24], [25]], JS and performance [[26], [27], [28]], the effect of SE on students' motivation, achievement, and adjustment [[29], [30], [31], [32]], JS and motivation [33,34], and SE and burnout [35,36]. Teachers should have pedagogical knowledge of teaching practice and “an awareness of their legal obligation to students and the educational community” [37]. In studies of teachers' SE and JS, the two variables have been shown to be essential factors to examine [38,39]. They may reduce the impact of several factors on teachers' implementation of curriculum since instructors who had high levels of them were more likely to adhere to their goals despite setbacks and to find creative solutions to problems that arise, hence, their SE and JS can mediate the effect of different factors on their curriculum implementation. Despite the significance of teachers' JS and SE to curriculum implementation, empirical studies of their relationships were rare [40]. To this point, it became significant to examine the effect of teachers’ SE and satisfaction on curriculum implementation.

Given the gap in the literature and the importance of internal and external factors to teachers' curriculum implementation, it turned out to be crucial to explore the effect of school culture on TCCI and the mediation role of internal factors, SE and JS, on this effect. This study introduced TPR, TTR, and TSR as factors that can affect TCCI in the Algerian context, from a teacher's perspective. In addition, this study introduced SE and JS as mediator variables that can mediate the effect of the above factors on TCCI. Teachers need more information about this impact so they can better participate in curriculum implementation. The current research is intended to fill this void and provide fresh perspectives to the literature by investigating the link between teachers' personal and professional circumstances and their effect on curriculum delivery. This research may help policymakers make more informed decisions on the deployment of various resources to promote teachers' dedication to curriculum implementation in an EFL context. This may provide cutting-edge research on EFL curriculum implementation, which is of considerable importance to schools and governments throughout the world. The most recent findings not only confirm the findings of previous research but also enhance the understanding of the relationship between school culture, teachers' SE, and JS. This research provided unique insights into the implementation of the EFL curriculum in Algeria, a developing country about which little is known.

## 2 Literature review

### 2.1 Social cognitive theory

This study explored the relationship between school culture, teachers' SE, and TCCI. These three main components of the study include environment, personality, and behavior. Social Cognitive Theory (SCT) highlights the triadic and asymmetric link between the features of environment, personality, and behavior that influence the performance of an individual [6]. Consequently, effects do not need to be proportional or instantaneous [6]. People take personal responsibility and manage life's complexity and uncertainties on their own. A person's environment, personality, and performance all play a role in the judgments they make about the most efficient means of achieving their goals. These elements contributed to the individuals who exist today.

In this study, school culture is primarily based on the relationships between teachers-principals, teachers-teachers, and teachers-students. The school culture is the surrounding environment that shapes agents' beliefs and performance [41,42]. These beliefs, which are SE and JS factors in this study, direct the resilient person toward favorable outcomes and away from those that might be detrimental, whereas behavior is TCCI. SE is "the idea that one has the capacity and skills to achieve one's objectives" [6] and JS is the degree to which an individual derives emotional contentment from his or her employment [[43], [44], [45]]. TCCI is the tutors' attachment to the curriculum policies and program implementation [46]. Throughout the goal-setting, strategy-planning, and outcome-anticipation processes, a person's underlying set of beliefs influences their behavior. In the triadic model, the SCT provided sophisticated ideas of interaction elements in order to describe and anticipate the fluid fluctuations of social life. Individuals were not only reactive to environmental stimuli but also proactive and capable of self-regulation, according to this a-genetic sociocognitive approach [47]. Consequently, individuals may be considered both "products" and "producers" of their surroundings [48].

Using SCT, this study looks at how culture, as an environmental factor, and SE and JS, as personal factors, affect teachers' commitment to curriculum. The research focuses primarily on the direct and unidirectional impacts of personal and environmental variables on the SCT. In other words, it investigated whether school culture (as an environmental element) significantly influenced the commitment of middle school teachers in Algeria to apply the curriculum. It also investigated how school culture impacts the commitment of Algerian middle school teachers to curriculum implementation via the mediation of personal variables (SE and JS).

### 2.2 The relationship between school culture, job satisfaction, and self-efficacy with curriculum implementation

Curriculum implementation has been defined as the process of delivering a curriculum to students to encourage learning while attempting to convey knowledge, behaviors, and attitudes [49]. Overall, it exhibited that teachers should be the primary source of concern for educational success in order to realize the curriculum's goals. TCCI refers to tutors' attachment to changes in policies and program implementation [46]. This was referred to as teachers' investment in their own and external resources to implement the change. This may enhance teachers' knowledge and orientation [50].

Herscovitch and Meyer (2002) [51] classified TCCI into three dimensions: effective commitment to change, continuance commitment to change, and normative commitment to change. Some researchers have investigated TCCI in various ways. Peng (2016) [52] explored the Chinese teachers' perspectives on TCCI. 23 participants were involved in the study with different experiences. School factors influenced the various perspectives of the participants regarding TCCI as indicated in the study. Thien and Adams (2019) [53] examined the correlation between the distributive leadership of school leaders and teachers' affective commitment to change. The study's sample group consisted of 531 Malaysian primary teachers. The results presented a significant positive relationship between the two variables, in which participative decision-making, leadership support, leadership supervision, and cohesive team leadership influenced teachers' affective commitment to change. Yu et al. (2020) [54] studied the commitment change of three English language tutors. The findings showed that the commitment level varies between four levels: committed passionate, committed compromiser, undecided, and uncommitted. The ﬁndings also demonstrated that such commitment change incorporated ongoing relations between SE, outcome expectations, professional autonomy, and social support provided by schools. In their study, Pham et al. (2023) [55] investigated the beliefs and experiences of elementary school educators in Vietnam during the initial stages of implementing a novel curriculum. The qualitative data analysis revealed that teachers expressed favorable opinions regarding the new curriculum, highlighting its numerous strengths and timely implementation. The teachers demonstrated a comprehensive grasp of the ideas and made diligent efforts to implement the new curriculum. They emphasized several of the curriculum's initial benefits for both students and themselves.

For many decades, school culture has been a subject of study. Loukas & Murphy (2007) [56] referred to this concept as the environment, culture, resources, and social networks surrounding a school. In other words, each school was characterized by its own distinct culture, which was based on the members' shared values, norms, and beliefs, thereby, teachers’ performance was influenced by that culture. School culture was known as “the basic assumptions, norms and values, and cultural artifacts that are shared by school members, which influence their functioning at school” [57]. These assumptions, norms, values, and cultural artifacts shaped the rapport between school agents, i.e., the principal, teacher, and student. In this way, the relationship between these agents formed a prominent component that represented that type of school culture [58]. This study defined school culture as the liaison between principal-teacher, teacher-teacher, and teacher-student. Literature has advocated the role that school culture plays in terms of the relationship between the aforementioned agents in determining the implementation of changes [59]. Bryk et al. (2010) [60] emphasized that cooperation between agents to achieve school goals and curricular coherence can lead to improving the school. Hulpia et al. (2009) [61] also confirmed the effect of the relationship between agents when they argued that students can improve when school leaders and tutors devote their effort and abilities to their learning progress. Price (2011) [59] found that the rapport between principals and tutors affected their commitment. He asserted:

One way to improve learning in schools is to focus on improving the relationships between principals and their staff, which produce satisfied, committed, and therefore more effective teachers. The benefits of trust and affective ties are central to this relationship process (69).

Min (2019) [9] examined the effect of these rapports on teachers' agency in curriculum reform autonomy implementation in South Korea. The study's sample consisted of 605 teachers at the elementary level. The results presented show that principal-teacher and teacher-teacher rapport affect teachers' agency towards curriculum autonomy implementation in schools and classrooms. Zhang and Liu (2013) [62] demonstrated that teachers who believed that an authoritative relationship with students would improve students' learning progress were resistant to implementing the new curriculum. According to Ravindran (2018) [58], school culture is a significant factor that can either positively increase or negatively decrease a teacher's improvement. That is to say, school culture automatically affects teachers' personal traits, such as a sense of identity, insights, actions, and their abilities to carry out their teaching practices, which were developed socially within the context of schools' culture [48]. For instance, previous research found that the support teachers receive from principals, colleagues, and students is indispensable for curriculum implementation [8]. Clayback et al. (2023) [63] confirmed that teachers who held more favorable initial opinions of the curriculum exhibited greater implementation. The relationship between teacher stress and perceptions of center climate and implementation was not constant. Lynch et al. (2023) [64] confirmed that when they demonstrated that the transitory nature of daily routines and a work environment characterized by continuous turnover of educators and administrators might result in the obscurity of teachers' efforts towards bringing about curriculum.

Positive school cultures built upon positive rapport between teachers, school principals, and students could sustain trust and collaboration for the teacher, which can thrust forward curriculum implementation. Aliyyah et al. (2023) [65] indicated that the effectiveness of the curriculum will mostly depend on the assistance and cooperation of all parties involved. Price, (2011) [59]. confirmed a strong relationship between high levels of trust and commitment and satisfaction among school members. Brezicha et al. (2015) [8], moreover, looked at how principals' leadership influences teachers’ different viewpoints on teaching as well as the flexibility of curriculum implementation alongside the support systems that led to greater curriculum implementation, thereby producing satisfied, committed, and effective teachers who felt positive and satisfied toward their job.

JS has been defined as the degree to which an individual derives emotional contentment from his or her employment [[43], [44], [45]]. Teachers who possess satisfaction from teaching generally love the surrounding environment and find that it provides them with positive sorts of engagement and involvement [66]. In addition, providing the necessary pedagogical and curriculum support is critical for effectively implementing a foreign language curriculum, such as English [67]. In this vein, literature advocates for the importance of JS. Teachers' JS has been shown to have a pivotal role in education since the quality of education is related to the tutors' quality and efficiency [68]. Research, for example, showed that JS may affect teachers' agency to teach due to the central role that acts in directing teachers' commitment, self-development, and creativity [69]. Skaalvik & Skaalvik (2011) [70] also presented that JS affects teachers' well-being since satisfied teachers are less prone to anxiety and burnout. Ostad et al. (2019) [71] found that JS influenced EFL teachers' self-expectations and duties and was affected by outside factors. However, Samadi et al. (2020) [72] did not find a positive correlation between JS and EFL teachers' burnout and intention to leave. This was due to the high level of JS among the EFL instructors, which reduced burnout and the intention to leave. Empirical findings confirmed that JS was positively associated with school culture and the working environment. According to Feng and Angeline (2010) [73], JS indicated the teachers' value judgments about their perspectives and expectations about the teaching effort they dispatched and the achievements they reached. Weasmer and Woods (2002) [74] also argued that JS was a vital factor that affected teachers' work commitment, advancement, and creativity, thus ensuring the stability of teaching as well as teachers' organizational commitment and effectiveness. Researchers suggested that satisfied teachers were more likely to be effectively committed to their jobs, allowing them to work at higher levels of performance [75]. Other studies on teacher job dissatisfaction, on the other hand, found that factors like a lack of career improvement opportunities, low motivation, poor working conditions, or fruitless communication with principals were prone to cause dissatisfaction [76]. Teacher turnover at such high levels will lead to ineffective results in terms of school efficiency as well as poor teaching and learning behaviors. Teachers’ relationships with principals, colleagues, and students were independent predictors of their sense of belonging, without them, teachers will leave due to a lack of commitment as a result of feeling unsupported and dissatisfied with their jobs [77]. The later claim emphasized the necessity of forming an atmosphere of mutual trust and respect across all school groups. With that said, social relationships with principals, colleagues, parents, and students contributed to a better sense of belonging, which led to increased JS.

Teacher's SE was another factor that may influence JS. Originating from social cognitive theory, SE has been defined as beliefs in the capabilities and skills to accomplish the desired aims [6]. Tschannen-Moran & Woolfolk Hoy (2001) [78] explained teachers' SE in terms of their beliefs about their abilities to identify the application and accomplishment of learning outcomes despite any challenges. The engagements and behaviors that teachers took throughout the process of teaching may be linked to their anticipations of their performance and their views of their ability. Supporting that, some researchers indicated that, compared to EFL teachers with low SE, EFL teachers with high SE are likely to devote their attention, energy, and skills in the pursuit of reaching their goals [79,80]. In this regard, SE has been considered significant for teachers to accomplish the intended objectives of curriculum implementation [24,80,81]. There was a growing body of research investigating the EFL teachers' SE about different factors affecting curriculum implementation. Caprara et al. (2003) [23] have revealed that teachers' SE beliefs have an essential role in affecting and sustaining teachers' commitment to school and JS. Efficacy beliefs have been positively linked to a set of aspects, such as higher levels of teacher job commitment, effective teaching practices, and higher academic achievement [82]. Imants and Zoelen (1995) [83] added that teachers' SE improved a solid commitment to the profession as well as cooperative relationships with colleagues and students, all of which contributed to the development of an inspiring and loaded teaching and learning environment. That is, SE and beliefs both shape and are shaped by an individual's behavior and environment [84]. He affirmed that teachers with a high sense of efficacy were considered to display more investment in teaching, set higher goals, put in more effort to attain those goals, and persevere when faced with impediments. Accordingly, teachers' satisfaction with their performance had a significant effect on their practices' success and, hence, on their students' academic achievement [85].

Gorozidis and Papaioannou (2011) [86] emphasized that highly self-efficacious teachers had a favorable attitude toward curriculum implementation. In other words, teachers who have a high sense of SE exhibit outstanding teaching abilities in terms of curriculum implementation, such as employing engaging teaching activities, establishing a positive learning atmosphere, and implementing a fitting evaluation system. They are more committed to their professional development and were more likely to have a positive influence on their teaching practices and students’ performance to effectively guide them in achieving their learning goals through successful curriculum implementation [78,87]. To summarize, any curriculum that did not align with teachers' beliefs about teaching would be less likely to be implemented, resulting in educational failure. Indeed, commitment to curriculum implementation would be enhanced by increasing teachers' sense of SE and JS would enhance commitment to curriculum implementation.

### 2.3 The present study

**Fig. 1:**
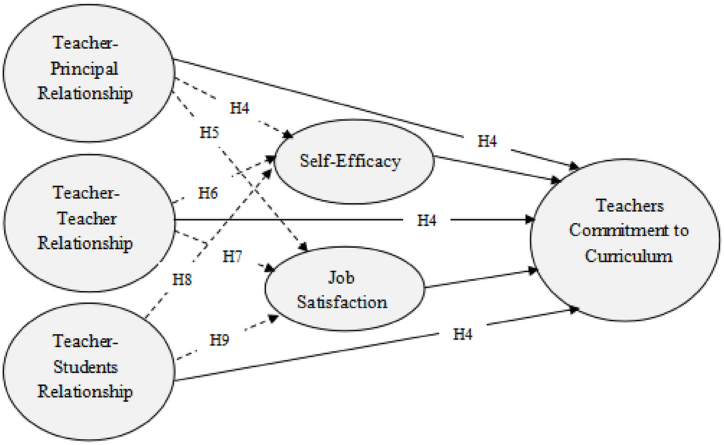
Hypothesized model of the study.

The selection of variables was based on their perceived significance in the education sector, as prioritized by the Algerian government. The Algerian government is currently striving to uphold high-quality interactions among school personnel while also ensuring that teachers effectively engage with their competencies and dispositions. Since President Abdelmadjid Teboun assumed the country's highest office, Algeria has recently given importance to teaching the English language in its educational institutions. The incumbent leader has introduced a strategy to promote cooperation among educational participants in order to attain optimal results in the English syllabus and linguistic education. This study aimed to investigate the effect of school culture on TCCI as well as the mediating role of SE and JS on the effect of school culture on TCCI. Two research questions guided this study: (a) Does school culture have a significant effect on TCCI? And (b) Do SE and JS have a mediating effect on the relationship between school culture and TCCI? Based on the literature presented above, some hypotheses were formulated. Furthermore, Fig. 1 shows the conceptual framework of the study, which visually represents the connection between the independent, dependent, and mediating variables under investigation. The independent variables consist of the TPR, the TTR, and the TSR. The mediating variables in this study are SE and JS. TCCI is the dependent variable. The black arrows depict the direct effect, while the dashed arrows represent the indirect effect. The following are study hypotheses.

H1 TPR has a positive and direct effect on the TCCI.

H2 TTR has a positive and direct effect on the TCCI.

H3 TSR has a direct and positive effect on TCCI.

H4 TPR has a significant indirect effect on TCCI via SE.

H5 TPR has a significant indirect effect on TCCI via JS.

H6 TTR has a significant indirect effect on TCCI via SE.

H7 TTR has a significant indirect effect on TCCI via JS.

H8 TSR has a significant indirect effect on TCCI via SE.

H9 TSR has a significant indirect effect on TCCI through JS.

## 3 Methodology

The primary objective of this study was to investigate the effect of school culture on TCCI as well as the mediating role of SE and JS on the effect of school culture on TCCI. This study employs a quantitative approach. The adoption of a cross-sectional research design allows for the collection of data from a diverse group of participants, facilitating the exploration and measurement of interactions between factors. The authors are a group of international researchers from China and Algeria. Their identities encompass a broad spectrum, including assistant professors and one Ph.D. student. They consist of researchers of all genders, including females and males. None of these influenced the study's scope and objective selection, nor did they impact the research methodologies used to achieve the study's goals. In order to examine the effect of school culture on TCCI as well as the mediating role of SE and JS on the effect of school culture on TCCI in a developing country, specifically Algeria, its education stakeholders are doing their best to promote the implementation of the EFL curriculum. A quantitative approach primarily centered around a cross-sectional research design was chosen to gather viewpoints from a substantial number of Algerian participants.

### 3.1 Participants

English language instructors from urban and rural public middle schools in Algeria participated in the current study. English teachers were recruited using a purposive sampling technique, which involved selecting individuals from various public middle schools in Algeria. Purposive sampling means picking units on purpose based on certain criteria that are important and relevant to the study [1,2]. The selection process was based on the participants' geographical proximity. The researchers sought to achieve a balance in the representation of teachers from rural and urban schools. Rural and urban regions in Algeria exhibit a disparity in teaching methods, contributing to this gap. Disparities exist in technology provision and utilization, instructional quality, academic achievement, support for schools and educators, school atmosphere, and student competency.

The questionnaire was sent to a total of 547 EFL teachers. They were selected from public middle schools in Algeria and contacted through both emails and by teachers' friends as volunteers for this study at their schools. The reason for utilizing both emails and friends lies in the geographical distance between researchers and participants, which necessitates either sending them emails or being contacted by acquaintances of the researchers. This facilitated researchers in obtaining a substantial number of participants, maximizing their sample size. In addition, there is a significant abundance of English as a Foreign Language (EFL) instructors in middle schools in Algeria. Nevertheless, the researchers were unable to contact all of them. They made a concerted effort to reach out to all of them via email and acquaintances of the researchers. The researchers have friends residing in several regions across Algeria. These friends are also Ph.D. researchers and are well-acquainted with the educational institutions located near their respective residences. They made a concerted effort to reach out to a substantial number of teachers. However, due to various factors, such as the unavailability of certain teachers, especially female teachers, and conflicting schedules with the researchers' acquaintances, the latter was unable to establish contact with all of them. In this regard, the researchers and their friends contacted the maximum number of EFL teachers at Algerian middle schools in rural and urban areas, whether males or females. The permission was obtained through a letter of informed consent. The sample was reduced to 533 teachers as there were those who did not provide full responses to the questionnaire. Hence, only fulfilled questionnaire forms were taken into account. In this regard, the final sample of the study is 533 English language teachers. The study's sample consists of 286 male instructors and 247 female teachers (M = 1.649). Teachers in the sample ranged in age from 27 to 52 years old (M = 2.901). There were 205 teachers between the ages of 27 and 31, 111 teachers between the ages of 32 and 36, 126 teachers between the ages of 37 and 41, and 91 teachers between the ages of 42 and older.

### 3.2 Research instrument

The questionnaire was divided into two sections. The first section was about participants' demographic information. The second section was about the six factors pertaining to the study's focus. The present research's instrument consisted of 49 items. The school culture scale consisted of eleven items split over three sub-scales: TPR, TTR, and TSR. These sub-scales were taken from Min's research (2019) [9]. The questionnaire's contents are closely aligned with the current research objectives and school culture factor, which enhances the potential for generalizing the results. In addition, the questionnaire is aligned with the same school culture dimensions: TPR, TTR, and TSR, which correspond to the current study model and hypotheses. Min (2019) [9] meticulously developed and validated the survey used in a previous investigation, with no reported ethical concerns. This approach is used in the present investigation to ensure the preservation of ethical integrity. The TPR and TSR scales each consisted of four items, whereas the TTR scale consisted of three items. This research utilized the SE measure developed by Hoy and Woolfolk (1993) [88]. The scale consisted of ten items. Furthermore, the JS scale was the scale developed by Evers et al. (2000) [89]. It had 10 items. The TCCI scale consisted of eighteen items derived from Herscovitch and Meyer's scale (2002) [51].

The scales Min (2019) [9] for school culture, Hoy and Woolfolk (1993) [88] for self-efficacy, Evers et al. (2000) [89] for job satisfaction, and Herscovitch and Meyer (2002) [51] for teachers' commitment to curriculum implementation were adopted as they are without any modification in the number of items. When scales are used in the same way they were intended, it helps the researchers increase the reliability of the study. It demonstrates that the study is consistent with accepted academic norms. The questionnaire's contents are closely aligned with the current research objectives and the four factors: school culture, self-efficacy, job satisfaction, and commitment to curriculum implementation, which enhance the potential for generalizing the results. Furthermore, the questionnaire utilized in previous investigations conducted by Min (2019) [9] for school culture, Hoy and Woolfolk (1993) [88] for self-efficacy, Evers et al. (2000) [89] for job satisfaction, and Herscovitch and Meyer (2002) [51] for teachers' commitment to curriculum implementation has been meticulously developed and validated, with no reported ethical concerns. This means the data has been verified for validity and reliability. This also demonstrates that the study focused on the proper variables in the questionnaire. This approach is used in the present investigation to ensure the preservation of ethical integrity.

Table 1 illustrates the scale item number. All the questionnaire items were rated on a 5-point Likert scale and in an ascending order of weighting: strongly disagree, disagree, neutral, agree, and strongly agree. The questionnaire was written in English since it targeted English teachers and was distributed from March 2021 to June 2021 to get the maximum number of participants in different provinces in Algeria.

**Table 1:** Questionnaire factors and items.

Scale Factors	Sub-factors	Number of Items	Examples
School Culture (11 Items)	TPR	4	● Teachers actively interact with principals ●The principal supports teachers in a way that creates an environment in which they feel comfortable working
	TTR	3	●Teachers cooperate with each other for school events ●Teachers do not tend to identify and exploit their co-workers’ weaknesses
	TSR	4	●Teachers understand the characteristics of their students well ●Teachers get along well with their students
SE		10	●The amount a student can learn is primarily related to family background ● If students are not disciplined at home, they aren't likely to accept any discipline ●If I try hard, I can get through to even the most difficult or unmotivated students
JS		10	Indicate the extent to which you feel satisfied or dissatisfied with your job: ●The degree to which you can personally develop or grow in your job ●The prospect of a raise
TCCI		18	●This change serves an important purpose ●It would be too costly for me to resist this change ●I feel a sense of duty to work toward this change

Before conducting the main research, four experts specializing in middle school administration and English teaching in Algeria provided feedback and ideas on the instrument's surface validity. The four experts were impressed with the scale's contents and recommended conducting pilot testing before the main study. 83 English instructors from public middle schools in Algeria participated in the pilot testing. Cronbach's alpha (α) was found to be 0.872, 0.791, 0.805, and 0.823, respectively, and the square root of α (0.934, 0.889, 0.897, and 0.907), respectively. This proved the validity and reliability of the research instrument [40,90,91] and permitted the main study to be conducted. The instrument was then sent to the study sample as stated above (n = 547), and full-answered versions of the questionnaire were considered (n = 533).

### 3.3 Data analysis

In order to analyze the study's data, numerous statistical programs were utilized. Descriptive statistics and exploratory factor analysis (EFA) were conducted using SPSS 22. Confirmatory factor analysis (CFA) was performed using JASP. Smart PLS was employed to determine SEM, which incorporated all pertinent coefficients, and evaluate direct and indirect effects among the study variables. Due to the importance of accurate data input to the success of this research, great care was taken in its execution. Each statistical method for every variable or hypothesis was explained in detail in the results section.

## 4 Results

### 4.1 Structural model

The factorial validity was examined to assess the validity of the scale. The KMO obtained in this study (KMO = 0.853) was higher than the values recommended by previous studies [90]. BST was significant (X2 = 4752.744, p ≤ 0.001). Hence, the normal distribution of data with multiple variables was affirmed. These results showed that the questionnaire was appropriate for factor analysis [90,92]. The most likely number of variables to match the data was six. Initial EFA with Eigenvalues for 49 items revealed a six-factor structure. One item was excluded from the TCCI factor due to loading less than 0.40, while the remaining items loaded higher than 0.40. In this regard, the final instrument items were 48, distributed among six factors: TPR (four items) with a factor load range between 0.59 and 0.78, TTR (three items) with a factor load range between 0.52 and 0.65, TSR (four items) with a factor load range between 0.55 and 0.61, SE (ten items) with a factor load range between 0.53 and 0.64, JS (ten items) with a factor load range between 0.58 and 0.72, and TCCI (seventeen items) with a factor load range between 0.57 and 0.79. Confirmatory factor analysis was also executed to confirm the research instrument items, and all loading values came higher than 0.53, and all factor loadings were statistically significant at p < 0.01.

Table 2 explains the item loading, Cronbach's alpha (α), composite reliability (CR), and discriminant validity values for each component, which were suitable and acceptable for this measurement [[93], [94], [95]]. Moreover, all Average Variance Extracted (AVE) values were higher than.50, indicating a good approximation of validity [90,96].

**Table 2:** Item loading,reliability construct and discriminent validity.

Factor	Item	Loading	α	CR	AVE	TPR	TTR	TSR	SE	JS	TCCI
						Discriminant validity					
TPR	Item1	0.78	0.769	0.802	0.633	**0.796**					
	Item2	0.63									
	Item3	0.75									
	Item4	0.59									
TTR	Item1	0.55	0.804	0.810	0.721	0.601	**0.849**				
	Item2	0.65									
	Item3	0.52									
TSR	Item1	0.59	0.741	0.783	0.624	0.566	0.700	**0.790**			
	Item2	0.61									
	Item3	0.57									
	Item4	0.55									
SE	Item1	0.56	0.800	0.805	0.643	0.532	0.612	0.708	**0.802**		
	Item2	0.58									
	Item3	0.60									
	Item4	0.61									
	Item5	0.64									
	Item6	0.63									
	Item7	0.55									
	Item8	0.62									
	Item9	0.53									
	Item10	0.59									
JS	Item1	0.70	0.827	0.859	0.753	0.689	0.567	0.590	0.757	**0.868**	
	Item2	0.68									
	Item3	0.63									
	Item4	0.72									
	Item5	0.65									
	Item6	0.61									
	Item7	0.66									
	Item8	0.58									
	Item9	0.62									
	Item10	0.59									
TCCI	Item1	0.69	0.871	0.880	0.766	0.550	0.710	0.613	0.599	0.760	**0.875**
	Item2	0.71									
	Item3	0.59									
	Item4	0.72									
	Item5	0.58									
	Item6	0.77									
	Item7	0.65									
	Item8	0.67									
	Item9	0.79									
	Item10	0.57									
	Item11	0.68									
	Item12	0.61									
	Item13	0.75									
	Item14	0.60									
	Item15	0.62									
	Item16	0.63									
	Item17	0.66									
	Item18	0.74									

As a casual comparative research design, the current study model employed quantitative techniques. The results of this study supported assessing both the direct effects of predictor variables on independent variables and the indirect effects of predictor variables through mediation variables on independent variables. The study model's fit indices revealed that it was a suitable model to use in determining the relationships among the study variables. By using the following criteria: CMIN(X2) = 3231.100, DF = 1613, AIC = 51105.359, BIC = 51970.147, CFI = 0.903, TLI. = 0.910, GFI = 0.909, AGFI = 0.900, NFI = 0.904, RMSEA = 0.071. The factor loading revealed that all things in the measurement model for each indicator had a relatively high loading. All factor loadings were highly significant due to matching all criteria values [2].

SEM was used to test and estimate the direct and indirect effects between the study's variables. All of the items were significant at a 0.01 level on their latent factors, and all of the factor loading was acceptable and supported the current study model. As such, all study hypotheses have been accepted, as shown in Table 3, and supported by the theoretical conceptions as follows: There is a positive and direct effect of TPR on TCCI (estimate = 0.381, SE = 0.053, Z = 5.100, p ≤ 001), there was a positive and direct effect of TTR on TCCI (estimate = 0.233, SE = 0.049, Z = 4.635, p ≤ 001), and there was a positive and direct effect of TSR on TCCI (estimate = 0.197, SE = 0.041, Z = 3.967, p ≤ 001).

**Table 3:** Direct effect of independent variables on dependent variables.

Hypotheses	Direct effect	Estimate	SE	Z value	P value	Decision
H_1_	TPR→TCCI	0.381	0.053	5.100	<0.001	Supported
H_2_	TTR→TCCI	0.233	0.049	4.635	<0.001	Supported
H_3_	TSR→TCCI	0.197	0.041	3.967	<0.001	Supported

**Fig. 2:**
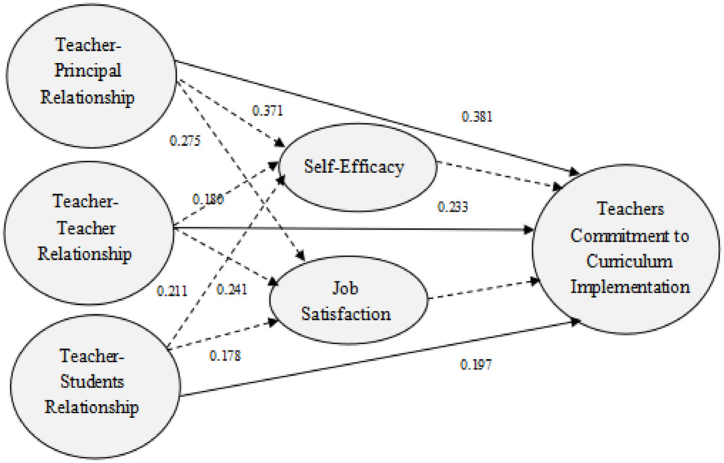
The structural model of the study.

To test the mediation of TATCR, Preacher and Hayes's (2008) [97] method was used, and p-values of indirect effects were gained through bootstrapping with 5000 resamples [98]. The results, as shown in Table 4 and Fig. 2, referred to the significant indirect effect of TPR on TCCI via SE (Estimate = 0.371, SE = 0.053, Z = 5.043, p ≤ 001), significant indirect effect of TPR on TCCI via JS (Estimate = 0.275, SE = 0.047, Z = 4.111, p ≤ 001), significant indirect effect of TTR on TCCI via SE (Estimate = 0.180, SE = 0.038, Z = 3.409, p ≤ 001), significant indirect effect of TTR on TCCI via JS (Estimate = 0.241, SE = 0.050, Z = 4.680, p ≤ 001), significant indirect effect of TSR on TCCI via SE (Estimate = 0.211, SE = 0.044, Z = 3.905, p ≤ 001), and significant indirect effect of TSR on TCCI via JS (Estimate = 0.178, SE = 0.035, Z = 3.399, p ≤ 001). Results substantiated that TPR, TTR, and TSR had a partial effect on TCCI via SE and JS.

**Table 4:** Indirect effect of independent variables on dependent variables.

Hypotheses	Indirect Effect	Estimate	SE	Z value	P value	Decision
H_4_	TPR→ SE →TCCI	0.371	0.053	5.043	<0.001	Supported
H_5_	TPR→ JS→ TCCI	0.275	0.047	4.111	<0.001	Supported
H_6_	TTR→ SE→ TCCI	0.180	0.038	3.409	<0.001	Supported
H_7_	TTR→ JS→ TCCI	0.241	0.050	4.680	<0.001	Supported
H_8_	TSR→ SE→ TCCI	0.211	0.044	3.905	<0.001	Supported
H_9_	TSR→ JS→ TCCI	0.178	0.035	3.399	<0.001	Supported

## 5 Discussion

The current study, using social cognitive theory, investigated the relationship between external factors, teachers' internal factors, and TCCI. A study looked at correlations between external factors (TPR, TTR, and TSR) and TCCI, as well as the role that internal factors (SE and JS) play in the relationship between those external factors and TCCI. These relationships were derived from the previously explained social-cognitive theory. Hence, an assessment of the relationship between variables was conducted.

The study revealed the positive and direct effect of TPR on TCCI. The results corroborated Brezicha et al.'s (2015) [8] findings that a strong relationship between teachers and principals, based on leaders' understanding of individual teachers' opinions and philosophies, could enhance the spread of reform information among tutors and support effective implementation of the reform by teachers. Previous studies have shown that leadership plays a crucial role in determining the implementation and outcomes of changes [99]. The adoption of innovative approaches was positively impacted by the backing of educational leaders [[100], [101], [102], [103]]. School leaders in Algeria can gain a better understanding of the obstacles to TCCI implementation by interacting with teachers about their concerns and providing assistance based on the instructors' requirements [7,104].

According to the data, there seemed to be a substantial positive effect of TTR on TCCI. Due to the significance of everyday interactions, several studies have explored how TTR influences teachers' abilities to innovate in the classroom. This is because everyday contacts are such a crucial component in shaping a school's culture [58,105]. This research gave credibility to the concept that excellent teacher relationships promote more academic flexibility for educators, both in terms of the curriculum they choose to teach and the schools and classrooms in which they do so. Instructors who were change agents were more willing to collaborate with their peers to improve their daily routines [106]. Most instances have proven this true. The amount of social support instructors get from their peers has a substantial effect on their readiness to implement innovations in the classroom [107]. However, Pitt (2010) [108] showed that less experienced instructors were less likely to employ innovative pedagogical practices when they interacted hierarchically and authoritatively with their more experienced colleagues. This decreased the likelihood that experienced educators would embrace these practices. Teachers should strengthen their relationships, interactions, and collaboration to facilitate curriculum implementation.

The results showed a significant effect of TSR on TCCI. Quality relationships with teachers profoundly impact students' academic achievement and the social, emotional, and behavioral health of their classroom communities [13,109,110]. However, the findings of this study seemed to infer that when teachers establish great relationships with their students, they are more likely to conduct the implementation in their classes in full. According to Zhang and Liu (2013) [62], teachers who believed that establishing an authoritative relationship with their students would hinder their academic advancement were unwilling to use the new curriculum. Students, who are more susceptible to the negative effects of school, may benefit enormously from pleasant, polite, empathetic, and persuasive interactions between students and instructors [111,112]. Hence, teachers may work to increase the level of interaction and relationship with their students in such a way that the classroom may create a calm and motivated environment that allows the implementation of the curriculum.

It was revealed that TPR, TTR, and TSR indirectly affect TCCI through SE and JS. In other words, instructors' SE and JS played a mediating role in the effect of school culture (TPR, TTR, and TSR) on TCCI. Consistent with Min's (2019) [9] findings, SE mediated the effect of school culture (TPR and TSR) on instructors' autonomy to implement the curriculum in South Korea. Min (2019) [9] found no evidence to support the idea that teacher-teacher interactions impacted classroom-level curricular implementation autonomy via teachers' SE. There was also evidence that the school environment impacted the JS when they were instructing [[113], [114], [115]]. The results provided support for past studies indicating that satisfied teachers mediate the relationship between a positive school atmosphere and school effectiveness [114]. According to Bakotic (2016) [66], teachers who like their professions think they have several opportunities to contribute to the communities in which they work. Teaching is a profession that may be extremely difficult on one's mental and emotional health, despite the fact that it can be quite rewarding. Members of the school community influenced teachers' contributions and performances, actively shaping and spreading the school's culture as a social environment variable.

## 6 Limitations and future research directions

Despite the researchers' best efforts to design the study well, it still has limitations that warrant caution when interpreting its findings. The study could address the relationship between school culture, self-efficacy, job satisfaction, and teachers’ commitment to curriculum implementation, which was not dealt with before. Prior research may have explored these variables singly or in combinations of two, but a complete approach that includes all four variables concurrently could reveal unique insights into their interconnections and cumulative impacts on one another. The detailed analysis may show nuances that were missed by studies that merely looked at one factor. By demonstrating a theoretical connection between these variables, the study can pave the way for further investigation. It can highlight potential cause-and-effect relationships and pave the way for longitudinal research, which might shed light on the interplay between different factors over time. These findings could inform the development of forecasting models that predict how changes in one factor, such as school culture, may impact another, such as the effectiveness of a new curriculum.

The study included a few factors: school culture in terms of TPR, TTR, TSR, SE, JS, and TCCI. Therefore, these factors may not apply to those affecting TCCI. Future studies, instead, can delve into more factors. The study instrument's validity and internal consistency were confirmed using Cronbach's alpha, CR, and AVE. Therefore, this may not fully depict the validity and internal consistency of the scale. Future research on the current study's topic may use predictive validity in addition to construct validity. This study did not consider demographic variables such as age, education, and gender. The general public's willingness to support school culture, SE, JS, and TCCI in Algerian schools may be better measured by incorporating demographic information into future studies. English teachers from primary and secondary schools were not included in the study population. The study population exhibits a disparity in terms of gender and age among participants. As a result, future research endeavors could incorporate participants' gender and age variables to further investigate their impact on teachers' implementation of the curriculum. Research on how the curriculum is implemented at various academic levels is strongly recommended for future researchers. Researchers in this study only analyzed the direct effects of TPR, TTR, and TSR on TCCI and their indirect effects via SE and JS, educational attributions will be further explored in subsequent investigations. It may or may not be useful to reflect here on the participants' views on how the curriculum was implemented based on a self-reported approach (survey). In prospective research on this subject, the use of interview and classroom observation data may increase teachers' perceptions of curriculum implementation.

## 7 Conclusion and implications

This study is among the few studies that investigated the relationship between school culture, teachers' SE, teachers' JS, and TCCI. In light of the UNESCO assessment that placed Algeria at the bottom of Arab nations in terms of education quality, we gave evidence, in accordance with social cognitive theory, that there were factors that influence teachers' commitment to the curriculum. The results showed that school culture in terms of TPR, TTR, and TSR affects EFL TCCI. These findings suggested a number of different courses of action for policy, research, and practice regarding EFL education and the interaction between teachers, principals, and students over the next several years. A primary objective for the government should be to strive toward the planned improvement of teachers' relationships with school leaders and students, as well as the provision of the necessary support needed to raise teachers' sense of SE and pleasure in the workplace. It was highly recommended that Algerian policymakers investigate the idea of establishing new guidelines to better structure the relationship between school administrators and teachers. It was also advised that school administrators strive for flexibility in their interactions with teachers and among teachers so that teachers are motivated and willing to engage with principals and their colleagues. Similarly, it was recommended that teachers urge students to collaborate with them and maintain constant communication. This may foster students' confidence in their teachers and their willingness to engage with them. The findings also revealed that school culture, as measured by TPR, TTR, and TSR, indirectly affects EFL TCCI through SE and JS. The greater the instructors' SE and JS, the greater their potential contribution to teaching effectiveness. Among the most successful techniques for increasing teachers' SE and JS include encouraging them to learn more about curriculum implementation, dealing with the school's circumstances and environment, and cooperating with their administrators and peers. Teachers may feel more confident and at ease when they participate in exchanges that do not put them in the limelight.

### 7.1 Ethics statement

Informed consent was gathered from all participating students. Confidentiality was maintained by not requesting names or any other information that would identify the students involved. The subjects were informed of their right to withdraw from the investigation at any time.

### 7.2 Disclosure of potential conflicts of interest

The authors declare that they have no known competing financial interests or personal relationships that could have appeared to influence the work reported in this paper.

## Funding

The authors did not receive support from any organization for the submitted work.

## Data availability statement

Data will be made available on request.

## CRediT authorship contribution statement

**Azzeddine Boudouaia:** Writing – original draft, Visualization, Resources, Project administration, Data curation, Conceptualization. **Abdo Hasan AL-Qadri:** Writing – review & editing, Validation, Software, Methodology, Investigation, Formal analysis. **Asma Houichi:** Writing – review & editing, Resources, Formal analysis, Data curation. **Salma Diafi:** Writing – review & editing, Resources, Data curation, Conceptualization.

## Declaration of competing interest

The authors declare that they have no known competing financial interests or personal relationships that could have appeared to influence the work reported in this paper.
